# Adaptation and Linguistic Validation of Angioedema PROMs in Latvian for Assessing Recurrent Angioedema

**DOI:** 10.3390/jcm14041375

**Published:** 2025-02-19

**Authors:** Lāsma Lapiņa, Adīne Kaņepa, Maksims Zolovs, Thomas Buttgereit, Nataļja Kurjāne

**Affiliations:** 1 Institute of Oncology and Molecular Genetics, Statistics Unit, Riga Stradiņš University, LV1007 Riga, Latvia; adine.kanepa@gmail.com (A.K.); maksims.zolovs@rsu.lv (M.Z.); natalja.kurjane@rsu.lv (N.K.); 2 Center of Clinical Immunology and Allegrology, Pauls Stradiņš Clinical University Hospital, LV1002 Riga, Latvia; 3 Allergic Diseases Diagnosis and Treatment Center, LV1003 Riga, Latvia; 4 Institute of Life Sciences and Technology, Daugavpils University, LV5401 Daugavpils, Latvia; 5 Institute of Allergology, Charité—Universitätsmedizin Berlin, Corporate Member of Freie Universität Berlin and Humboldt-Universität zu Berlin, 10117 Berlin, Germany; thomas.buttgereit@charite.de; 6 Fraunhofer Institute for Translational Medicine and Pharmacology ITMP, Immunology and Allergology, 12203 Berlin, Germany

**Keywords:** angioedema, patient-reported outcome measures (PROMs), quality of life, disease activity and control, linguistic validation of PROMs

## Abstract

**Background:** Angioedema (AE) is a localized, non-pitting swelling affecting subcutaneous and/or submucosal tissues. Despite varying underlying mechanisms, AE significantly impacts patients’ quality of life (QoL), which is closely linked to disease activity and control. **Objectives:** This study aimed to translate and linguistically validate the angioedema activity score (AAS), angioedema control test (AECT), and angioedema quality of life (AE-QoL) questionnaires into Latvian, and to use these validated tools to assess disease activity, control, and quality of life within the study population. **Methods:** PROMs, including the AECT, AAS, and AE-QoL, underwent a standardized linguistic validation process. Patients with hereditary angioedema (HAE), mast cell-mediated angioedema (AE-MC), and angioedema of unknown origin (AE-UNK) were recruited from two separate studies conducted at Riga Stradiņš University. **Results:** We enrolled 41 participants (90.2% women) with a mean age of 46.3 years. AE-MC was the most common (63.4%), followed by HAE (19.5%) and AE-UNK (17.1%). The mean AAS score was 15.8, with no significant differences regarding AE type, gender, or age. The mean AECT score was 8.29, revealing significant gender differences (women: 7, men: 13.5). The AE-QoL total score was 45.5, with significant gender differences in most domains. Strong correlations were found between AE-QoL scores and both AAS and AECT, highlighting the impact of both disease activity and control on QoL. **Conclusions:** The Latvian adaptation of the AAS, AECT, and AE-QoL questionnaires effectively assesses AE activity, control, and disease-related QoL. Our study reveals poor disease control, underscoring the need for tailored interventions and regular PROM evaluations, with the Latvian version of the AE-QoL questionnaire identifying five distinct domains compared to four in the original version.

## 1. Introduction

Angioedema (AE) is a non-pitting, self-limited, localized swelling that involves the subcutaneous and/or submucosal layers of tissue. It affects the face, lips, neck, extremities, oral cavity, larynx, and/or gut, lasting from a few hours up to several days [1]. AE may present independently or in conjunction with wheals (hives), depending on the underlying endotype. Often chronic, AE is characterized by recurrent swellings that can vary in frequency. The anatomical site of AE can influence its impact: it may cause disfigurement, functional impairment, pain, or airway obstruction. Laryngeal involvement poses a potentially life-threatening risk, while intestinal angioedema can be severely painful and often mimics acute abdominal conditions [1,2]. Consequently, the unpredictable nature of the disease, combined with the severity of edema attacks and the potential for life-threatening episodes, substantially impacts patients’ quality of life (QoL).

AE can be induced by various etiological factors and pathophysiological mechanisms [3]. The most common type of recurrent AE is mast cell mediator-induced angioedema (AE-MC), which arises from the release of mediators from mast cells that increase vascular permeability, leading to tissue edema. AE-MC may be associated with wheals (hives) and/or pruritus, though this is not always the case. It can be triggered by allergic reactions, pseudoallergic reactions, or anaphylactic reactions, or may present as a form of chronic urticaria (chronic inducible or chronic spontaneous urticaria). Notably, chronic urticaria is predominantly the primary cause of recurrent AE-MC [2,4].

- Allergic reactions causing AE typically result from immunoglobulin E (IgE)-mediated type I hypersensitivity reactions [5,6].
- Pseudoallergic reactions exhibit clinical symptoms similar to type I reactions; they are distinct from IgE-mediated allergies. Several mechanisms underlying pseudoallergies have been described in the literature:

Complement activation-related pseudoallergy (CARPA) [7,8,9,10,11].

Non-IgE-mediated anaphylactoid reactions [12].

- AE as a manifestation of urticaria (acute, chronic) [4,13,14,15].

A rarer pathophysiological mechanism of AE is bradykinin-mediated angioedema (AE-BK), which results from excessive bradykinin production due to dysregulation of the contact pathway, or decreased catabolism, such as in ACE inhibitor-induced AE, leading to edema. AE-BK can be hereditary or acquired [1,16,17,18,19].

Additionally, AE can result from various medications (e.g., ACE inhibitors, hormones, NSAIDs, etc.), leading to what is termed drug-induced angioedema (AE-DI). The underlying mechanisms of AE-DI vary with the medication and its specific adverse effects:

- AE induced by angiotensin-converting enzyme (ACE) inhibitors [20,21,22,23,24,25];
- AE induced by nonsteroidal anti-inflammatory drugs (NSAIDs) [26,27,28,29];
- AE secondary to hormone replacement therapy (HRT) [30];
- AE induced by recombinant tissue plasminogen activator (rtPA) [31,32,33,34];
- AE induced by dipeptidyl peptidase-IV (DPP-IV) inhibitors, or gliptins [35,36].

AE due to intrinsic vascular endothelium dysfunction encompasses types of HAE associated with genetic mutations leading to vascular endothelium dysfunction and altered endothelial permeability regulation [37,38,39,40,41].

AE of unknown origin (AE-UNK) is classified as such when no identifiable pathophysiological mechanisms, as previously described, can be determined.

Regardless of the underlying pathogenic mechanisms, AE significantly impacts patients’ daily functioning and QoL. This impact is not only due to the physical deterioration associated with the severity of AE attacks but also due to the psychological burden of the condition. AE can cause substantial short-term disability during episodes and lead to persistent anxiety between attacks. Its unpredictable nature may affect patients’ daily decisions, leading them to avoid travel, specific hobbies, or social opportunities. Additionally, concerns about starting a family or other social and relational challenges may arise. It is well-documented that psychiatric conditions, such as anxiety and depression, are more prevalent among patients with AE [42,43,44]. Although many studies have focused on the psychological health of patients with HAE, it is likely that AE from other pathogenic mechanisms similarly contributes to significant psychological distress.

To assess the psychological burden of AE, evaluate patients’ QoL, and monitor disease activity and control, which are crucial for developing effective treatment strategies, international guidelines recommend utilizing patient-reported outcomes (PROMs) at each specialist visit. Validated tools, such as the angioedema activity score (AAS), angioedema quality of life questionnaire (AE-QoL), and angioedema control test (AECT), are available in multiple languages and should be employed for this purpose [4,17]. To streamline the collection of assessment data, reduce the reliance on paper questionnaires, and save time during appointments, several mobile applications have been developed by experts in the field. Notable examples include HAE TrackR and CRUSE Control [45]. These applications provide a convenient and efficient solution for both patients and clinicians, facilitating real-time data entry and monitoring while enhancing the overall management of AE. By leveraging these digital tools, healthcare providers can improve patient care and optimize the assessment process during clinical visits.

Until now, standardized questionnaires such as the AAS, AECT, and AE-QoL were unavailable in Latvia for clinical and research purposes. Therefore, this study aimed to translate these questionnaires into Latvian and conduct thorough linguistic validation to ensure their accuracy and cultural relevance. This process involved meticulous translation and expert assessments to verify the integrity of the translated versions. Additionally, the study sought to use these validated instruments to gain a comprehensive understanding of disease activity, control, and QoL among Latvian patients with AE. This approach not only enhances the local assessment of AE and highlights related challenges but also supports the development of targeted management strategies tailored to the specific needs of this cohort.

## 2. Materials and Methods

### 2.1. Patients

This study involved two distinct patient cohorts recruited from Pauls Stradiņš Clinical University Hospital and the Allergic Diseases Diagnosis and Treatment Center, both part of ongoing PhD research projects at Riga Stradiņš University.

The first cohort consisted of patients with AE-MC identified through the hospital’s electronic information system using the International Classification of Diseases (ICD) code L50.8. Participants were randomly selected, aged 18 years or older, and invited to join the study. Inclusion criteria were based on diagnostic guidelines by the EAACI/GA^2^LEN/EuroGuiDerm/APAAACI [4] for CSU, which required the exclusion of other potential causes of urticaria and/or AE. This ensured the cohort’s specificity. Patients with other types of urticaria and/or AE were excluded. This cohort included 19 patients with AE-MC, all of whom participated in the questionnaire study.

The second cohort comprised patients with AE from Pauls Stradiņš Clinical University Hospital and the Allergic Diseases Diagnosis and Treatment Center who had consulted allergists or immunologists and were coded with ICD D84.1. The cohort included 8 patients with genetically confirmed HAE, who tested positive for mutations in the SERPING1 gene; 7 patients with AE-MC; and 7 patients with AE-UNK, all of whom were tested for 6 mutations (SERPING1, F12, PLG, ANGPT1, KNG1, and MYOF) and found to be negative.

All participants were thoroughly briefed on the study’s objectives, methodologies, and procedures, and provided informed consent. Ethical approval for the study was granted by the Ethics Committee of Riga Stradiņš University.

During scheduled appointments, 41 patients underwent a comprehensive evaluation using standardized assessments: AE-QoL questionnaire, AECT, and AAS.

Two patients under the age of 18 participated in the study: the 17-year-old completed the questionnaires independently, while the 9-year-old was assisted by their parents. In both cases, informed consent was obtained and signed by the patients and their parents.

### 2.2. Questionnaires Used in the Study

Patient-reported outcome measures (PROMs) employed in this study included the AE-QoL, AECT, and AAS questionnaires.

- The AAS is a daily diary-type questionnaire that prospectively assesses angioedema activity over four weeks, providing a valid measure of disease activity.
- The AECT evaluates disease control retrospectively, with a recall period of either four weeks or three months. It consists of four questions, and its scores range from 0 (no control) to 16 (complete control), with a cut-off of 10 indicating controlled disease. The minimal clinically important difference (MCID) is three points, signaling meaningful improvement.
- The AE-QoL is a 17-item tool designed to assess health-related quality of life (HRQoL) retrospectively over four weeks. Results can be presented as a total score or as four domain scores, ranging from 0 to 100. Higher scores indicate greater HRQoL impairment, and the MCID of six points reflects significant HRQoL improvement [46].

All questionnaires underwent a meticulous linguistic validation process to ensure the reliability and cultural appropriateness of the instruments used to assess the impact of AE on the Latvian population.

The linguistic validation process of the PROMs involved several standardized steps to ensure equivalence across different language versions:

1. Forward Translation: Two independent forward translations of the PROMs from the source to the target language were generated by a professional translator and a healthcare professional (HCP)—an immunologist treating AE patients. Both translators were native speakers of the target language and bilingual in the source language.
2. Reconciliation: The two forward translations were reconciled into one version by an HCP.
3. Back-Translation: An independent back-translation of the reconciled version from the target language into the source language was performed by another professional translator, who was a native speaker of the source language and bilingual in the target language.
4. Review by Original Authors/Developers: The back-translations were reviewed by the original authors/developers of the PROMs, who compared them against the original versions and provided comments and suggestions for changes.
5. Adjustment: The target language version was refined based on the comments and suggestions from the first developer review. Further back-translation of the adjustments was conducted to ensure accuracy.
6. Cognitive Debriefing: The final consensus version from step 5 was tested with five patients, encompassing both genders (4 females, 1 male) and a broad age range (32 to 61 years), all of whom were native speakers of the target language and affected by the disorder. Under supervision, patients completed the PROMs and were then interviewed to evaluate the clarity of the wording, identify any challenging or distressing terms, verify accurate paraphrasing, and ensure proper understanding of the concepts.
7. Final Adjustment: The target language version was refined based on the feedback from the cognitive debriefing interviews.
8. Second Developer Review: The original authors and developers conducted a final review of the cognitive debriefing feedback and the subsequent adjustments to the target language version, thereby concluding the linguistic validation process.

The structured translation process and cognitive debriefing interviews were meticulously documented using standardized forms.

### 2.3. Statistical Analysis

Data distribution was assessed using normal Q-Q plots and the Shapiro-Wilk test. Continuous variables are expressed as mean ± standard deviation or median with the first and third quartiles, while categorical variables are expressed as total number and percentage.

For comparisons between groups with independent observations, one-way ANOVA and independent samples *t*-tests were employed for normally distributed data with homogeneity of variances. For non-normally distributed data, the Kruskal-Wallis H test and the Mann-Whitney U test were utilized. Spearman’s correlation was applied to determine associations between parameters such as AECT, AAS, AE-QoL, and demographic data.

Internal consistency of the measurement instruments was assessed using Cronbach’s alpha, with values of 0.70 or higher deemed acceptable. Factor analysis with Varimax rotation was applied to enhance the interpretability of the factor structure by maximizing the variance of squared loadings while maintaining orthogonal factors. Statistical analysis was performed using Jamovi v. 2.3.28. A significance level of *p* < 0.05 was considered statistically significant.

## 3. Results

### 3.1. Sample Characteristics

We enrolled 41 participants (90.2% female), with ages ranging from 9 to 80 years (mean age 46.3 ± 16.5 years). The most represented age group was 50–59 years (24.4%). AE-MC was diagnosed in 26 patients (63.4%), HAE in 8 patients (19.5%), and AE-UNK in 7 patients (17.1%). The gender comparison results may be influenced by the limited representation of male participants in the study. Table 1 provides a detailed summary of the demographic data.

### 3.2. PROMs in the Study Population

#### AE Activity and Control

The mean AAS questionnaire score in the study population was 15.8 ± 31.7 with no statistically significant associations found between AAS scores and AE type (*p* = 0.129), gender (*p* = 0.149), or age (*p* = 0.189).

The mean AECT score was 8.29 ± 4.76. There were no statistically significant differences in AECT scores between AE type (*p* = 0.657) or age (*p* = 0.148). However, a significant difference in AECT score was observed between genders, with women (median = 7.00, IQR 4–12) scoring lower than men (median = 13.5, IQR 13–14.5), U = 21.5, *p* = 0.022, r = 0.709 (high effect size). A majority of patients (n = 26, 63%) had poor AE control (AECT < 10), while the remaining patients (n = 15, 36.6%) exhibited well-controlled AE (AECT ≥ 10).

A significant strong negative correlation was found between AAS and AECT scores (r = −0.550, df = 39, *p* < 0.001).

The mean AE-QoL total score was 45.5 ± 24.5. There were no statistically significant associations between AE-QoL scores and AE type (*p* = 0.888) or age (*p* = 0.569). However, there was a significant difference in AE-QoL total score between genders, with women (median = 47.8, IQR 31.6–61) scoring higher than men (median = 14.7, IQR 11–15.4), U = 7.43, *p* < 0.001, r = 2.09 (high effect size).

We analyzed the structural composition of the AE-QoL domains in our study population, finding differences from the original AE-QoL structure, which includes four domains (Functioning, Fatigue/Mood, Fears/Shame, and Nutrition) [46]. The Latvian version identified five nuanced domains: Fears/Shame/Functioning, Sleep/Fatigue, Nutrition, Physical Activities/Mood, and Side Effects (Table 2).

To estimate the reliability of a composite score, we used Cronbach’s α calculations. For all items included in Domain 1, Cronbach’s α was >0.90, indicating very good internal consistency. For Domains 2 and 3, the included items had a Cronbach’s α > 0.70, indicating good internal consistency, except for item 11 in Domain 3, which had a Cronbach’s α of 0.649, showing slightly insufficient internal consistency among the domain items (Table 3). Since Domain 4 included only 2 questions and Domain 5 only 1 question, Cronbach’s α calculation formula was not applicable to these domains.

The comparison of domain structures between the original and Latvian versions of the AE-QoL questionnaire revealed several similarities. Specifically, Domain 2 in the Latvian version, which includes questions 6, 7, and 8, corresponds to the original Fatigue/Mood domain (questions 6, 7, 8, 9, 10). Domain 1, which includes questions 1, 3, 4, 9, 12, 13, 14, 15, and 16, aligns with the original Fears/Shame domain (questions 12, 13, 14, 15, 16, 17). Domain 3, consisting of questions 5, 10, and 11, is similar to the original Nutrition domain (questions 5 and 11). This adaptation underscores the importance of cultural and linguistic considerations in questionnaire design.

The analysis of the Latvian-specific domains of the AE-QoL questionnaire revealed the following:

- Fears/Shame/Functioning: This domain showed the highest impact with a median score of 52.8 (IQR 19.4–69.4). Significant gender differences were found, with women scoring higher (median = 58.33, IQR 33.33–75) compared to men (median = 5.56, IQR 4.17–9.03), U = 10.5, *p* = 0.006, r = 0.858 (high effect size). No significant differences were observed between AE types (*p* = 0.407) or age groups (*p* = 0.528).
- Sleep/Fatigue: This domain had a median score of 41.7 (IQR 0–75) for women and 0 (IQR 0–4.17) for men, with significant gender differences (U = 27.5, *p* = 0.039, r = 0.628 (high effect size)). There were no significant differences based on AE type (*p* = 0.666) or age (*p* = 0.143).
- Nutrition: This domain did not show significant differences based on AE type (*p* = 0.741), age (*p* = 0.570), or gender (*p* = 0.258).
- Physical Activities/Mood: The median score was 50.0 (IQR 0–50) for women and 0 (IQR 0–6.25) for men, with significant gender differences (U = 30.5, *p* = 0.045, r = 0.588 (high effect size)). No significant differences were found based on AE type (*p* = 0.741) or age (*p* = 0.924).
- Side Effects: This domain showed the least impact, with a median score of 25 (IQR 0–50). There were no significant differences based on AE type (*p* = 0.471), age (*p* = 1.0), or gender (*p* = 0.263).

These findings highlight the nuanced impact of AE on QoL, particularly across different domains and between genders. Table 4 and Table 5 provide a detailed summary of the PROM scores in the study population and the differences in questionnaire scores between genders.

The AE-QoL scores, which reflect patient well-being and are theoretically linked to disease activity and control, were analyzed in relation to the AAS and AECT questionnaire results. The observed correlations are summarized in Table 6 below:

These associations underscore the relationship between the impact of AE on various quality-of-life domains and disease activity and control, emphasizing the importance of comprehensive assessment tools in managing AE.

## 4. Discussion

In our study, we demonstrated the critical importance of evaluating AE to assess the course of the disease, facilitate therapeutic decisions, and ensure optimal patient care. Developing and applying specific evaluation instruments is essential not only at the regional level but also internationally. Unified questionnaires are necessary to conduct comprehensive studies on affected patients, evaluate regional and global trends, and identify differences related to AE. These questionnaires must be standardized, user-friendly, and culturally adapted to each population to ensure accuracy and relevance in diverse settings.

The Latvian version of the AE-QoL questionnaire identified five nuanced domains: Fears/Shame/Functioning, Sleep/Fatigue, Nutrition, Physical Activities/Mood, and Side Effects. These domains may differ from the original developers’ version [47] due to various factors, with cultural influences being paramount. Cultural context significantly shapes how patients perceive and articulate their HRQoL. While this adaptation enhances the relevance of PROMs for local populations, it also introduces challenges by altering the domain structure, which complicates data aggregation and comparison in international studies. For this reason, adhering to the original domains is preferable in the context of multinational research to maintain consistency and comparability across diverse populations [48,49,50,51,52].

It is well known that AE clinical manifestations can be similar despite being caused by different pathophysiological mechanisms [1,2,4,17,53]. This similarity complicates clinicians’ decision-making processes concerning diagnostic, therapy choices, and patient monitoring. In our study, we demonstrated that there are no statistically significant differences between the types of AE and PROM results. This finding justifies that there is no need to introduce separate disease assessment questionnaires for each type of AE. Instead, utilizing specific, internationally recognized questionnaires is sufficient. However, it is important to acknowledge that the imbalance in AE subtype prevalence could influence the interpretation of our findings. The smaller sample sizes for HAE and AE-UNK may have limited statistical power to detect significant differences between subtypes. It is important to note that PROMs are primarily intended to monitor disease-related outcomes and assess the need for treatment adjustments. These standardized tools are well-defined, do not impose an extra burden on patients and clinicians, and facilitate consistent and efficient disease assessment.

In our study, we found no statistically significant differences between age groups and the PROM results. However, the results could be compromised due to the small study population and uneven age group distribution, as most of our patients were over 40 years old. AE can affect individuals of all ages, and the skewed age distribution may have influenced our findings. The high percentage of female patients in the cohort may have further impacted the results, as gender differences could potentially affect the outcomes. Additionally, the study attracted more participation from patients whose disease is difficult to control with available treatment options, particularly in the non-HAE groups. This selection bias may have impacted the results, contributing to the unsatisfactory PROM outcomes observed [54,55,56,57].

We observed a predominance of female patients (37 [90.2%]) compared to male patients (4 [9.8%]), which aligns with findings from other studies [58,59] that also report a female predominance in the AE patient population. The literature attributes these differences, particularly in HAE and AE-MC, to the effects of estrogen on these forms of AE [30]. Additionally, we found that female patients, regardless of AE type or age, exhibited poorer disease control and QoL. This could be related to hormonal differences as previously mentioned, but it is also important to consider the potential impact of socioeconomic factors and patient selection biases [57]. These factors combined suggest a multifaceted interplay that warrants further investigation to better understand and address the gender disparities observed in AE outcomes.

The observed suboptimal disease control and QoL in our study population may be influenced by multiple factors [60,61,62,63,64,65]. A centralized healthcare system and limited access to specialized care in certain regions could impede effective disease management. Gaps in physician expertise regarding treatment strategies and experience with therapy prescription and monitoring may also contribute to inadequate care. Additionally, patient-related factors such as individual treatment preferences and adherence to prescribed therapies play a significant role. Insights from international studies suggest that variations in disease control and QoL among AE patients could be further attributed to disparities in treatment accessibility, healthcare infrastructure, and adherence to treatment guidelines across different regions [4,17]. Inconsistent access to advanced therapies, including monoclonal antibodies or small molecules, may impact disease management. Socioeconomic factors, cultural attitudes towards healthcare, and varying levels of patient education also influence treatment adherence and overall disease control. Furthermore, additional investigations are needed to explore how factors such as residence, comorbidities, concomitant medications, and socioeconomic conditions correlate with disease control and QoL outcomes as measured by PROMs. These factors were not addressed in the current study but are critical for a comprehensive understanding of the multifaceted influences on disease management and patient well-being. Addressing these gaps through targeted research and tailored healthcare policies has the potential to enhance patient care, refine treatment strategies, and improve outcomes across diverse populations. Future studies should prioritize larger, more diverse samples to validate the current findings and improve their generalizability, particularly to populations underrepresented in this study, such as males and other demographic groups. Additionally, given the dynamic nature of disease progression and treatment responses, patient-reported outcomes should be measured longitudinally. This would allow for the assessment of changes in symptoms, quality of life, and treatment effectiveness over time, providing deeper insights into the long-term impact of the disease and its management.

## 5. Conclusions

In conclusion, the translation and linguistic adaptation of the AAS, AECT, and AE-QoL questionnaires into Latvian have provided reliable and culturally appropriate tools for assessing AE activity, control, and QoL in the Latvian population. Our study revealed that the majority of patients experience poorly controlled disease and diminished QoL, with the Latvian version of the AE-QoL questionnaire identifying five distinct domains compared to four in the original version. These findings underscore the necessity of regular evaluation using these PROMs in clinical practice and highlight the need for tailored interventions to enhance disease management and patient outcomes.

## Figures and Tables

**Table 1:** Study cohort description.

Gender, n (%)	
Men	4 (9.8%)
Women	37 (90.2%)
Age (years), mean (standard deviation)	46.3 (±16.5)
Age group distribution, n (%)	
9–17	2 (4.9%)
18–24	3 (7.3%)
25–30	3 (7.3%)
31–39	5 (12.2%)
40–49	8 (19.5%)
50–59	10 (24.4%)
60–69	7 (17.1%)
70–80	3 (7.3%)
Distribution by angioedema type, n (%)	
Mast cell-mediated angioedema, n (%)	26 (63.4%)
Women	24 (58.5%)
Men	2 (4.9%)
Hereditary angioedema, n (%)	8 (19.5%)
Women	7 (17.1%)
Men	1 (2.4%)
Angioedema of unknown origin, n (%)	7 (17.1%)
Women	6 (14.6%)
Men	1 (2.4%)

**Table 2:** Latvian AE-QoL domain structure.

	Component					
	1	2	3	4	5	Uniqueness
15. I am ashamed to go out in public because of swelling episodes	0.835					0.2207
13. I am afraid of the sudden onset of angioedema	0.803					0.3015
4. Angioedema interferes with my social relationships	0.739					0.2175
16. I feel embarrassed by swelling episodes	0.710					0.2029
1. Angioedema interferes with my work	0.670					0.1793
3. Angioedema interferes with my leisure time	0.668					0.0777
12. The episodes of angioedema are burdensome for me	0.612					0.2110
14. I am afraid that the frequency of swelling episodes might increase	0.605					0.2463
9. Difficulties in concentrating	0.580					0.2111
8. I feel tired during the day because of my bad night sleep		0.850				0.1163
7. I wake up during the night		0.849				0.1227
6. Difficulties in falling asleep		0.772				0.3552
11. I have to limit my choices of food and/or beverages			0.934			0.1031
5. Angioedema interfere with my eating and drinking behavior			0.741			0.2149
10. I feel depressed			0.695			0.2055
2. Angioedema interferes with my physical activities				0.826		0.1402
17. I am afraid that the treatment of the angioedema could have negative long-term effects					0.814	0.2663
**Summary**						
**Component**	**SS Loadings**	**% of Variance**		**Cumulative %**		
1	4.68	27.52		27.5		
2	3.05	17.94		45.5		
3	2.51	14.75		60.2		
4	1.92	11.28		71.5		
5	1.45	8.55		80.0		

SS = sum of squares.

**Table 3:** Item reliability statistics.

**Component 1**		
Cronbach’s α 0.932		
**Item**	**Item-Rest Correlation**	**Cronbach’s α**
13. I am afraid of the sudden onset of angioedema	0.676	0.928
15. I am ashamed to go out in public because of swelling episodes	0.739	0.925
4. Angioedema interferes with my social relationships	0.819	0.920
16. I feel embarrassed by swelling episodes	0.714	0.926
1. Angioedema interferes with my work	0.768	0.923
3. Angioedema interferes with my leisure time	0.845	0.919
12. The episodes of angioedema are burdensome for me	0.783	0.922
14. I am afraid that the frequency of swelling episodes might increase	0.652	0.930
9. Difficulties in concentrating	0.761	0.923
**Component 2**		
Cronbach’s α 0.856		
**Item**	**Item-rest correlation**	**Cronbach’s α**
7. I wake up during the night	0.758	0.746
8. I feel tired during the day because of my bad night sleep	0.808	0.722
6. Difficulties in falling asleep	0.613	0.899
**Component 3**		
Cronbach’s α 0.814		
**Item**	**Item-rest correlation**	**Cronbach’s α**
5. Angioedema interferes with my eating and drinking behavior	0.704	0.703
10. I feel depressed	0.554	0.849
11. I have to limit my choices of food and/or beverages	0.752	0.649

**Table 4:** PROM scores in study population.

		Mean Score	Standard Deviation
**AAS**		15.8	31.7
**AECT**		8.29	4.76
**AE-QoL**			
**Total score**		45.5	24.5
**Scale domains and included items**		**Median score**	**IQR**
I—Fears/Shame/Functioning	1, 3, 4, 9, 12, 13, 14, 15, 16	52.8	19.4–69.4
II—Sleep/Fatigue	6, 7, 8	41.7	0–66.7
III—Nutrition	5, 10, 11	33.3	8.33–75
IV—Physical activities/Mood	2	50	0–50
V—Side effects	17	25	0–50

**Table 5:** Questionnaire score comparison between genders.

PROMs	*p*	Mean Difference	95% Confidence Interval		Effect Size
			Lower	Upper	
AAS7	0.576	2.67 × 10^−5^	−1.51 × 10^−5^	19.00	NA
AECT	0.022	−7.00	−11.00	−1.00	0.709
AE-QoL-Total	<0.001	35.5	10.29	66.18	2.09
AE-QoL-Fears/Shame/Functioning	0.006	47.22	13.89	72.22	0.858
AE-QoL-Sleep/Fatigue	0.039	41.67	1.52 × 10^−5^	75.00	0.628
AE-QoL-Nutrition	0.258	16.67	−8.33	66.67	NA
AE-QoL-Physical activities/Mood	0.045	50.00	2.30 × 10^−5^	50.00	0.588
AE-QoL-Side effects	0.263	9.47 × 10^−7^	−1.32 × 10^−5^	50.00	NA

NA = not applicable.

**Table 6:** Correlation analysis of AECT, AAS, and AE-QoL questionnaires.

		AAS	*p*-Value	AECT	*p*-Value
AE-QoL-Fears/Shame/Functioning	r	0.572	<0.001	−0.812	<0.001
AE-QoL-Nutrition	r	0.316	0.044	−0.464	0.002
AE-QoL-Physical activities/Mood	r	0.482	0.001	−0.647	<0.001
AE-QoL-Side effects	r	0.247	0.120	−0.312	0.047
AE-QoL-Sleep/Fatigue	r	0.369	0.018	−0.514	<0.001
AE-QoL-Total	r	0.526	<0.001	−0.815	<0.001

## Data Availability

The data that support the findings of this study are available on request from the corresponding author. The data are not publicly available due to privacy or ethical restrictions.

## Abbreviations

AAS7	Angioedema activity score over 7 days
AECT	Angioedema control test
AE	Angioedema
AE-BK	Bradykinin-mediated angioedema
AE-DI	Drug-induced angioedema
AE-MC	Mast cell mediator-induced angioedema
AE-QoL	Angioedema quality of life questionnaire
AE-UNK	Angioedema of unknown origin
CSU	Chronic spontaneous urticaria
DPPIV	Dipeptidyl peptidase-IV
HAE	Hereditary angioedema
HCP	Healthcare professional
HRQoL	Health-related quality of life
ICD	International classification of diseases
MCID	Minimal clinically important difference
rtPA	Recombinant tissue plasminogen activator
PROMs	Patient-reported outcome measures
QoL	Quality of life
